# Philanthropic sales in live-streaming shopping: The impact of online interaction on consumer impulse buying

**DOI:** 10.3389/fpsyg.2022.1041476

**Published:** 2022-10-13

**Authors:** Yusen Ye, Zhili Zhou, Huawei Duan

**Affiliations:** ^1^Institute for Disaster Management and Reconstruction, Sichuan University, Chengdu, Sichuan, China; ^2^School of Management, Xihua University, Chengdu, Sichuan, China

**Keywords:** philanthropic marketing, live-streaming shopping, empathy, online interaction, impulse buying

## Abstract

As philanthropic sales *via* live-streaming shopping have played an important role in alleviating the huge backlog of agricultural products during the outbreak of the COVID-19 pandemic, this paper aims to study how online interaction in philanthropic marketing exerts influence on consumer impulse buying behaviors. We empirically explore four major dimensions of online interactions in philanthropic live-streaming sales, i.e., the live streamers’ image, the herd effect of consumers, the responsiveness of sellers, and the mutual trust between consumers. The results reveal that the herd effect of consumers and the responsiveness of sellers could promote consumers’ empathy ability toward the growers of the products sold lively, whereas the live streamers’ image and the mutual trust between consumers have little effect on empathy promotions. Meanwhile, both the consumers’ empathy ability and the live streamers’ image positively affect consumers’ impulse buying behavior, which suggests a partial moderating role of consumers’ empathy ability. Lastly, by taking both social and business perspectives, we provide managerial implications for improving the effectiveness and efficiency of philanthropic live-streaming sales to alleviate social and economic pressure in emergencies.

## Introduction

During the outbreak of the COVID-19 pandemic in early 2020, agricultural and sideline products from Hubei Province in China were in deep stagnation due to the severe pandemic impact on sales channels, which seriously reduced the income of local farmers and slowed down the economic development of society. To open up the sales channels to other areas outside Hubei critically affected by the pandemic and alleviate social and economic pressure, the philanthropic sales mode of the e-commerce platform has become the main sales mode to solve the backlog problem of local agricultural products. In 2021, China’s live-streaming users have exceeded 635 million, wherein more than 38% watch philanthropic sales live and 27.8% watch agriculture-supporting sales live. With the rapid development of Internet surfing, live-streaming shopping has become a mainstream media channel for selling products. Given the unique characteristics, such as marketing innovation and real-time interaction, the live-streaming mode becomes easier to make consumers’ immediate and unplanned purchases. Accordingly, philanthropic marketing can enhance the output of agricultural and sideline products in Hubei and other places and increase consumer purchases intuitively, instantly, and in bulk. The relevant data show that all kinds of popular live streamers such as local official media and local leading cadres have raised the daily sales of agricultural products from Hubei for philanthropic broadcast up to 52,000 with an increase of 360.2%, and the live e-commerce market size has increased 121.5% year-on-year with the output as high as 961 billion RMB. It is recognized that philanthropic sales can effectively boost consumers’ willingness to purchase in live-streaming. However, due to the special nature of philanthropic behavior, what factors in live-streaming interaction will affect consumers’ purchase behavior need to be further explored.

Studies have shown that impulse buying accounts for up to 70% of consumer purchase behaviors (Zeng, 2006). Piron (1991) believes that impulse buying is related to the emotions of individual consumers and is an immediate, unplanned purchase resulting from consumers’ emotional responses to a specific stimulus. In the commercial context, scholars have studied impulse buying based on the mediating role of presence, from the perspective of the tripartite interaction between consumers and websites, consumers and online sellers, and consumers and consumers (Jiang et al., 2014; Chen et al., 2021). This is due to the fact that in a commercial environment, consumers pay for the hedonic experience, which is gained through in-person offline shopping and the pleasure of interacting with people in both directions. However, during the pandemic, the purchase motive of consumers participating in philanthropic live-streaming activities has changed, no longer principally to gain hedonistic experience but to help farmers in Hubei and other areas seriously affected by the pandemic to get rid of the stagnant sales plight of agricultural products. With increasing concern for the affected areas, and continuous perception of the urgent need for help, consumers’ assistance intentions for farmers increased significantly, forming a sense of empathy. This empathic ability derives from the psychology of personal sorrow and empathic concern (Lai et al., 2021), but not from hedonic experiences. Therefore, the immersive stimulus created by online interactions in the traditional commercial live marketing model may no longer have a moderating effect on impulse buying behavior in philanthropic live-streaming. Thus, it remains to study whether online interactions affect the formation of consumers’ empathy *via* philanthropic live-streaming, and then may have an impact on consumers’ impulse buying. In philanthropic marketing *via* live-streaming, live streamers involve various types such as popular live streamers and stars, local official media, local leading cadres, etc. In particular, cross-border live-streaming combining live streamer and star has achieved good sales results. Therefore, this paper classifies the type of “live streamer + star” as flow live-streaming (celebrities with a huge fan base), whereas the type of “official media + local leading cadre” is as official live-streaming. Research has shown that popular live streamers are better at mobilizing consumers’ active participation than stars, thus promoting consumers’ purchasing behaviors (Fei and Zhou, 2021). Then whether the emerging type of live streamers spawned by the philanthropic live-marketing model in the pandemic context influences consumers’ impulsive purchase behaviors has not been addressed in the current studies.

While the majority of live-streaming shopping research lay the focus on the sales of hedonic products in the commercial world, this paper innovatively explores the influence of online interaction on consumers’ impulsive purchase behavior in philanthropic live-streaming sales, which is motivated by the real case of the stagnation of agricultural and sideline products in the context of the COVID-19 pandemic. This is because in such an emergency context, the mediating effect that online interaction has shown on consumers’ impulsive purchasing behavior in philanthropic sales is typical and different, which worth further understanding. Moreover, to the best of our knowledge, the similarities and differences that how online interaction influences consumers’ impulsive purchase behavior in commercial and philanthropic live-streaming models have not yet been compared and analyzed.

With this research using the Stimulus-Organism-Response (S-O-R) model, we characterize the online interaction in philanthropic live-streaming sales from four dimensions of stimuli, i.e., the live streamers’ image, the responsiveness of sellers, the herd effect of consumers, and the mutual trust between consumers with dual perspectives of live streamer and consumer. We show the mediating role of empathy ability under the control of the live streamer type to explore the intrinsic influence mechanism of online interaction on consumers’ impulse purchases. The partial moderating effects of consumers’ empathy ability in both flow and official live-streaming modes have been compared and analyzed in detail. Based on our empirical results, we also provide management suggestions on how the philanthropic live-streaming sales model can promote consumer participation and enhance sales efficiency under the influence of emergencies, with the purpose of alleviating economic hardship.

## Related literature

As this research looks at how online interaction in philanthropic live-streaming sales influences consumers’ impulsive purchase behavior, the literature on online interaction, empathy theory, and impulse buying behavior is of relevance.

### Online interaction

Interaction refers to the initiators’ response to other participants. The term interaction encompasses several dimensions. From the perspective of existing studies, researchers have not yet reached a consensus on the division of the dimensions of interaction. Ku (1992) proposed six dimensions of interaction: immediate feedback, responsiveness, diversity of sources, communication links, equality of involvement, and ability to terminate. Zhao et al. (2014) divided the interaction into functional interaction, perceptual interaction, and process interaction. Xiong (2014) found that information quality interaction, network tool quality interaction, and service quality interaction together constitute online interaction. Dong et al. (2018) proposed four dimensions of online interaction based on a “storyline”: online sensory interaction, online emotional interaction, online entertainment interaction, and online behavioral interaction. Zhou and Zuo (2012) explored online interaction between consumers from the perspective of virtual communities. Liu et al. (2012) found that in the online shopping context, online interactions also involve herding behavior. Some studies found that in the current era of the new economy, the personal charisma of the live streamer, the high level of interactivity, and the quality of the content will lead consumers to make impulsive purchases (Jiang, 2019; Lu et al., 2021). In Koufaris view, the interaction between the website and the online consumer could stimulate the consumer’s interest in purchasing (Koufaris, 2002). In summary, online interaction can trigger impulse purchases, but how online interaction affects consumers’ impulse purchases under the philanthropic live-marketing model needs further study. Since the perspective of interactive subject participation has not yet been refined, given the special nature of philanthropic live-marketing and the uniqueness of online platforms, the following will explore the influence of four factors on consumers’ impulsive purchase intentions, namely, the live streamers’ image, the herd effect of consumers, the responsiveness of sellers and the mutual trust between consumers.

### Empathy theory

Empathy is the ability to perceive and understand the emotions of others with behavioral responses (Decety and Svetlova, 2012). Tucker (2016) explained empathy as an emotional ability to place the self in the situation of others. Some researchers argued the value of empathy marketing through emotional interaction, empathic interaction, and affinity interaction (Chen and Guo, 2021; Lin et al., 2021). Wang (2021) verified the effect of online microblogging interaction on social empathy. Some studies even pointed out that the combination of empathy theory and business models can better contribute to the growth of business value. Previous studies show that effective interaction increases the effect of empathy. In Hubei’s special philanthropic live-streaming sales campaign, which assists the sales of agricultural products in Hubei during the pandemic, how the interaction between live streamers and consumers as well as between consumers and consumers affects the empathy of pro-social consumers is one of the focuses of this paper.

### Impulse buying behavior

Impulse buying refers to unplanned and unconcerned regretful purchases from stimulating the audience users’ instant desire to buy (Gao, 2018). There are many studies investigating the factors that influence impulse buying. Xiong and Jing (2010) constructed a model of the factors influencing consumer impulse buying in terms of marketing stimuli, context, and personal characteristics. He (2013) developed on this basis, and the study concluded that individual traits, marketing stimuli, situational factors, and other factors are influential on consumer impulse buying. Based on the perspective of individual traits: Zhao et al. (2015) segmented products based on involvement and emotional factors and then researched customers’ impulse buying. Liu et al. (2017b) conducted a study corresponding to consumer emotions and impulse buying in terms of the visual appeal of the website pages to consumers and the ease of use of the website by customers. Based on the marketing stimulation perspective: Yin (2013) examined consumer impulse buying from an Internet promotional marketing perspective. Zhang and Li (2017) explored the intensity of consumer impulse buying from the perspective of different time-limited pressure and promotional offers. From the context perspective: Zhao et al. (2014) explored the reasons for stimulating consumers’ impulsive purchases from an online product display perspective. Zhu et al. (2017) found that two important factors influencing consumers’ impulse purchases were the quality of online user reviews and the reviewer’s rank. Hu (2015) has conducted a corresponding study based on the social interaction of views and behaviors. However, the underlying mechanism that drives consumers to perform impulse purchases in philanthropic live-streaming shopping waits for further empirical evidence.

## Materials and methods

### Theoretical model

**Figure 1:**
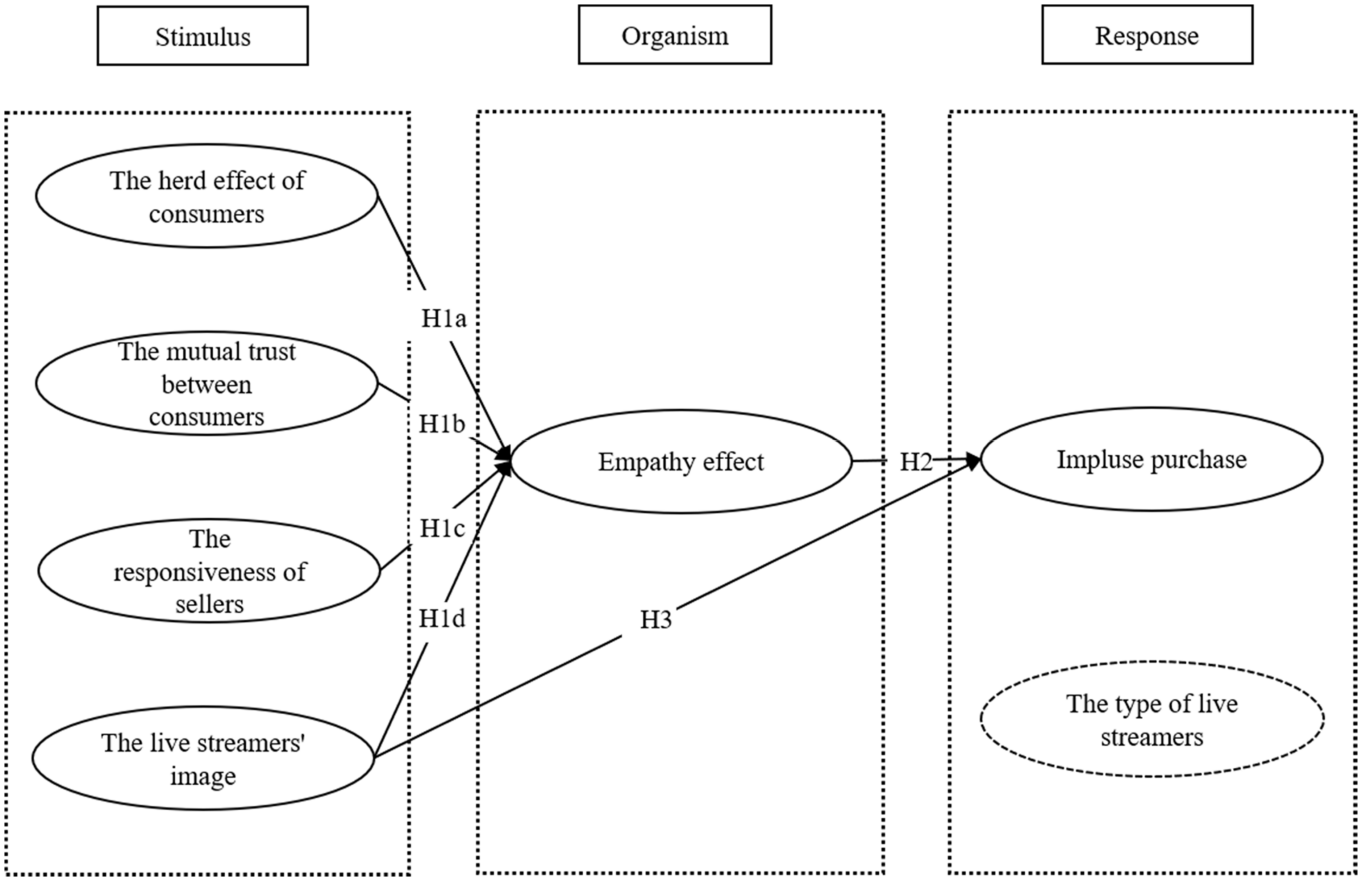
The theoretical model.

Previous studies have shown that online interaction affects consumer impulse buying. Based on the S (stimulus)-O (organism)-R (response) model, as shown in Figure 1, this paper takes live-streaming interaction in Taobao philanthropic sales as an external stimulus, the empathy effect generated by consumers as a mediating variable and the type of live streamer as a control variable, and finally the consumer impulsive purchase as a behavioral response. We then comprehensively explore the influence mechanism of online interactions on consumers’ impulsive purchases.

### Research hypotheses

#### Online interaction and empathy effect

There are very limited studies focusing on online interaction and empathy effect in live-streaming shopping, only Tan et al. (2020) pointed out that in interpersonal interaction, individual recognition and perception of others’ information will affect their emotional resonance. With the desire to provide assistance to the affected population and alleviate the economic hardship caused by the pandemic, the more intensive the interactions between consumers, live streamers, and other consumers are, the more likely the emotional resonance will produce. Therefore, this study puts forward the following hypotheses on the relationship between online interaction and consumer empathy effect under philanthropic live-streaming:

*H1*: Online interaction in live shopping positively correlates with the empathy effect of consumers.

We divide online interaction into four variables:

- The interaction between live streamers and consumers
- The effect of live streamers and responsiveness
- Interaction between consumers
- The herd effect of consumers and the mutual trust between consumers

Liu et al. (2017a) believe that individual users with social preferences are willing to share their consumption experiences on social networks. Moreover, some researchers point out that online reviews about products could influence consumers’ purchasing behavior (Zhao et al., 2020; Li et al., 2022). However, merchants often increase their business profits by adding comments that confuse the real experience of the commodities (Ding and Yu, 2022). Therefore, the following hypotheses are made:

*H1a*: The herd effect of online interaction is positively correlated with the formation of the consumer empathy effect.

*H1b*: The mutual trust of online interaction is negatively correlated with the formation of the consumer empathy effect.

*H1c*: The responsiveness of online interaction is positively correlated with the formation of the consumer empathy effect.

*H1d*: The online interaction effect of streamers is positively correlated with the formation of the consumer empathy effect.

#### Empathy effect and impulse buying

Many scholars have found that they have a positive relationship in the study of the empathy effect and consumer purchase (Chen et al., 2022; Jing et al., 2022). Nevertheless, in the public broadcast mode, whether consumer impulse purchase will be positively affected by the empathy effect remains to be studied. Therefore, this study proposes the following hypothesis:

*H2*: The formation of the consumer empathy effect positively correlates with consumer impulse buying.

#### Live streamers’ image and impulse buying

Previous studies have shown that “celebrity” attraction positively affects consumers’ purchases (Liu et al., 2020), while current live streamers with millions of fans have been given a “celebrity” halo. Therefore, this study puts forward the following hypothesis for the live streamers’ effect and consumer impulse buying:

*H3*: The effect of live streamers is positively correlated with consumer impulse buying.

### Research design

#### Questionnaire

As the proportion of live users using the Taobao live-streaming platform (i.e., the largest online C2C e-commerce platform in China) is as high as 68.5%, this study targets Taobao live-streaming users in the design of the questionnaire. Regarding the measurement of independent variables such as responsiveness, mutual trust, live streamers’ effect, and herding effect of online interaction, we refer to the scales introduced by Wu (2006), Tang (2007), Song and Zinkhan (2008), Zhang (2009) and Liu et al. (2012). Similar to Vijayasarathy (2002) and Jing and Yue (2005) survey on impulse buying, Yuan (2011) measurement of impulse buying intention, Davis (1983) measurement of the empathy effect, we form the final measurement scale (Table 1) with modest modifications based on the previous research scales, which also combines the characteristics of charitable live-streaming shopping during the COVID-19 pandemic.

**Table 1:** Measurement items and variable explanations.

Item	Description
Live streamers’ image F1	①When shopping *via* philanthropic live-streaming, I think the streamers I follow have extraordinary personalities.
	②When shopping *via* philanthropic live-streaming, I think the streamer has a wealth of information about the products for sale.
	③When shopping *via* philanthropic live-streaming, the products recommended by the streamer give me the desire to buy.
Responsiveness of sellers F2	①When shopping *via* philanthropic live-streaming, the streamer can respond to my questions on time.
	②When shopping *via* philanthropic live-streaming, the streamer’s response is closely related to my question.
	③When shopping *via* philanthropic live-streaming, the streamer is very happy to communicate with me.
Herd effect of consumers F3	①I shop *via* philanthropic live-streaming because I see my friends buying.
	②When shopping *via* philanthropic live-streaming, I like to buy products that have the most visitors or are praised by people.
	③When shopping *via* philanthropic live-streaming, the more buyers of the product, the more I want to buy.
Mutual trust between consumers F4	①When shopping *via* philanthropic live-streaming, I think the buyer’s comments are mostly spurious to confuse the authenticity of the use of the goods.
	②When shopping *via* philanthropic live-streaming, I think the buyer’s goods are shared not to optimize my purchase but for the merchant’s profitable marketing purposes.
	③When it comes to philanthropic live-streaming shopping, I think a 5% lousy review is more convincing than a 95% decent review.
Consumers’ empathy ability F5	①When shopping *via* philanthropic live-streaming, I think the live site will make me feel the depression under the Hubei pandemic.
	②When shopping *via* philanthropic live streaming, I think the live bullet comments will remind me of a similar experience.
	③When shopping *via* philanthropic live-streaming, I think I would think about the state of the pandemic in Hubei.
	④When shopping *via* philanthropic live-streaming, Slogans such as “I am three pounds fatter for Hubei” can make me feel enthusiastic.
Consumers’ impulse buying behaviors F6	①When shopping *via* the philanthropic live-streaming, I will find something that is not in the plan but I want to buy.
	②When shopping *via* the philanthropic live-streaming, I will buy some products that I did not intend to buy.
	③When shopping *via* the philanthropic live-streaming, I will buy without thinking.

#### Research method

This study employs AMOS 24 and SPSS 25 software to test and analyze the proposed model and hypotheses using structural equation modeling (SEM). Two main components of models are distinguished in SEM: the measurement model showing the relations between latent variables and their indicators, and the structural model describing potential causal dependencies between endogenous and exogenous variables.

Specifically, the equation of the measurement model is characterized as:


$$x=\Lambda_{x}\xi +\varepsilon$$

$$y=\Lambda_{y}\eta +\sigma .$$

In our research, x is a 12×1 vector of the observations for 4 exogenous latent variables consisting of attitude toward the live streamers’ image, the herd effect of consumers, the responsiveness of sellers, and the mutual trust between consumers, while y is a 7×1 vector that observes 2 endogenous latent variables, i.e., consumers’ empathy ability and consumers’ impulse buying behaviors. In terms of the exogenous latent variables, ξ is a 4×1 vector and η is a 2×1 vector. $\Lambda_{x}$ refers to the 12×4 factor loading matrix of x on ξ, $\Lambda_{y}$ refers to the 7×2 factor loading matrix of y on η, and ε and σ denote the error terms of the exogenous and endogenous variables, which cannot be explained by the latent variables.

Then, the equation of the structural model is expressed as:


η=Bη+Γξ+λ.

B is a 2×2 coefficient matrix of the interaction between endogenous latent variables, Γ is a 2×4 coefficient matrix that shows the influence of exogenous latent variables on endogenous latent variables, η and ξ refer to the vectors given by the measurement model, and λ shows the unexplained part of the model, such as confounding factors or residuals.

## Empirical findings

### Descriptive statistics

We target experienced consumers in Taobao philanthropic live-streaming purchases. Before the formal release of the questionnaire, a pre-survey was conducted to test the reliability and validity of the questionnaire design. The sample data of the questionnaire were collected through sharing links on social media as well as the professional questionnaire market. A total of 537 questionnaires were distributed with 496 pieces finally collected. The feedback of 33 of the respondents was excluded because they did not use Taobao live-streaming during the COVID-19 pandemic. Finally, 463 valid questionnaires were used for this research. The collection rate of valid questionnaires is 86%, which meets the requirement of a valid sample five times the measurement items. The results of demographic characteristics are shown in Table 2.

**Table 2:** Demographic description.

Demographic characteristics	Classification	Frequency	Percentage (%)
Gender	Male	208	44.92
	Female	255	55.08
Age	<25	43	9.29
	25 ~ 30	126	27.21
	31 ~ 35	179	38.66
	36 ~ 40	93	20.09
	≥40	22	4.75
Educational level	High school education and below	27	5.83
	Vocational College Degree	52	11.23
	Bachelor degree	320	69.11
	Master degree	58	12.53
	Doctor degree and above	6	1.30
Salary (CNY/Month)	<2000	67	14.47
	2001 ~ 4,000	92	19.87
	4,001 ~ 6,000	131	28.29
	6,001 ~ 8,000	107	23.11
	8,001 ~ 10,000	58	12.53
	≥10,000	8	1.73

Amongst the valid questionnaires, male respondents take a proportion of 44.92% and females take 55.08%, with a relatively balanced gender structure. Regarding the age structure, 9.29% are under 24 years old, 27.21% are between 25 and 30 years old, 38.66% are between 31 and 35 years old, 20.09% are between 36 and 40 years old, and 4.75% are over 40 years old. The sample population structure is consistent with that of Freshippo and Missfresh, which is reasonable. In terms of educational background, the proportion with a bachelor’s degree or above accounts for 82.94%, and others accounts for 17.06%. As for the income structure, over 51.4% of the respondents earn a salary between 4,000–8,000 RMB a month. The statistical characteristics of the sample population are consistent with the demographic characteristics of the industry analysis report for Chinese fresh e-commerce released in 2022 (iiMedia Report, 2022).

### Reliability and validity

In this research, reliability is tested by Cronbach’α. When Cronbach’α is less than 0.5, the reliability is poor; between 0.5 and 0.7, the reliability is normal. The reliability is good when Cronbach’α is between 0.7 and 0.9 and great when it is over 0.9. As can be seen from Table 3, the reliability of the entire questionnaire is 0.842 with the Cronbach’α coefficients of 6 latent variables all higher than 0.7. Therefore, the reliability of each scale suggests that the questionnaire design is reasonable.

**Table 3:** Results of reliability and validity analysis.

latent variable	Item	Factor load	Cronbach’α	KMO	CR	AVE
F1	Ab1	0.761	0.736	0.664	0.746	0.496
	Ab2	0.617				
	Ab3	0.727				
F2	Re1	0.823	0.833	0.714	0.835	0.629
	Re2	0.734				
	Re3	0.819				
F3	En1	0.681	0.764	0.676	0.774	0.534
	En2	0.806				
	En3	0.699				
F4	He1	0.833	0.793	0.691	0.800	0.573
	He2	0.777				
	He3	0.649				
F5	Em1	0.583	0.777	0.768	0.784	0.480
	Em2	0.766				
	Em3	0.773				
	Em4	0.629				
F6	Bu1	0.724	0.723	0.655	0.740	0.491
	Bu2	0.776				
	Bu3	0.587				

The validity of the sample data is tested using KMO (Kaiser-Meyer-Olkin) and Bartlett coefficients, factor load, CR (combined reliability), and AVE (mean–variance extraction). Table 3 shows that the KMO value of the entire questionnaire is 0.882, wherein the KMO values of 6 latent variables are all over 0.6, suggesting that the measurement scale is suitable for factor analysis. The exploratory factor analysis (EFA) and confirmatory factor analysis (CFA) show that the factor load of each measurement item is more significant than 0.5, the AVE is more significant than 0.4, and the CR is more significant than 0.7, indicating that the measurement variables have good convergence validity and internal consistency.

Because this study adopts the questionnaire survey method, the Harman single factor test method is used to test the homologous variance of data to prevent homologous variance. The results show that the maximum factor variance interpretation rate is 33.448%, less than 40%. Moreover, as demonstrated in Table 4, the square root value of AVE for each factor is greater than the absolute value of the correlation coefficient between the elements, implying good discriminant validity. Therefore, this study has no common method deviation, and data analysis can be carried out.

**Table 4:** Discriminant validity: Pearson correlation vs. AVE square root value.

	Factor1	Factor2	Factor3	Factor4	Factor5	Factor6
Factor1	**0.704**					
Factor2	0.641	**0.793**				
Factor3	0.574	0.446	**0.731**			
Factor4	−0.154	−0.188	−0.056	**0.757**		
Factor5	0.502	0.452	0.536	−0.044	**0.693**	
Factor6	0.471	0.317	0.485	−0.041	0.402	**0.700**

Diagonal numbers are AVE square root values.

### Model analysis

Structural equation modeling was selected as the test method to test this study’s research framework and hypotheses. Firstly, the total speculative hypothesis model is tested. Secondly, theoretical assumptions that do not pass are excluded. Finally, the control variable, i.e., the live streamers’ type, is introduced to analyze how online interaction affects consumer impulse buying mediated by empathy and how the live streamer effect affects consumer impulse buying. In this paper, Amos24.0 software is used for model fitting analysis. As shown in Table 5, the initial model fitting indexes all reach the standard values, and the model fitting effect is good. The hypothesis test results are shown in Table 6.

**Table 5:** Initial model fit test.

	$\chi^{2}$	df	$\chi^{2}/df$	AGFI	NFI	IFI	CFI	RMSEA	SRMR
**Standard**			<3	>0.9	>0.9	>0.9	>0.9	<0.06	<0.05
**Result**	346.390	140	2.474	0.899	0.905	0.941	0.941	0.056	0.0514

**Table 6:** Initial hypothesis test.

Hypothesis	Estimate	P	Result
H1a	0.443	^***^	**√**
H1b	0.052	0.278	
H1c	0.217	0.018	**√**
H1d	0.166	0.260	
H2	0.182	0.026	**√**
H3	0.501	^***^	**√**

*** *p* < 0.001.

**Figure 2:**
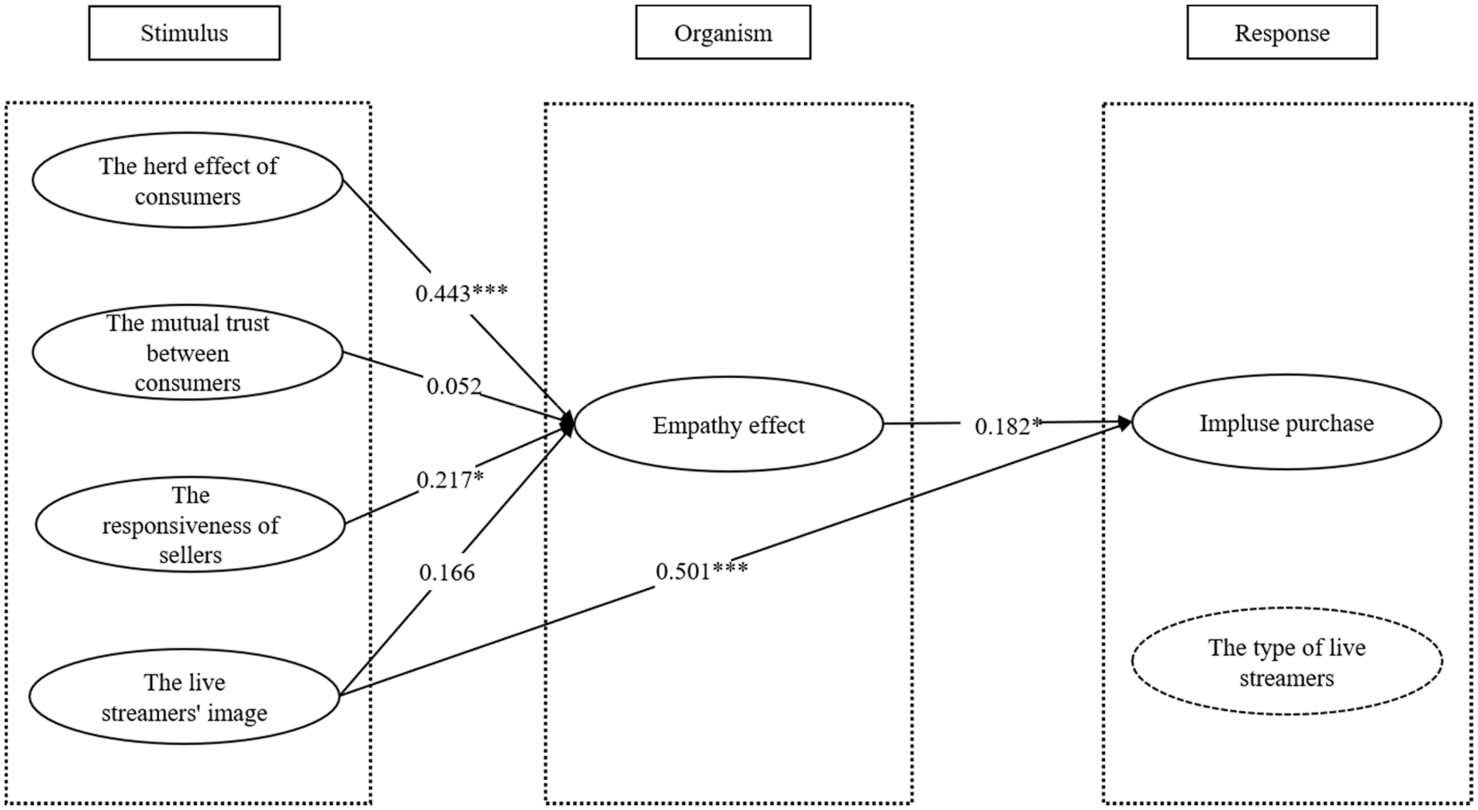
Path coefficient test.

It can be seen from Figure 2 and Table 6 that H1b and H1d are not valid. After eliminating the relationship, Amos software is used to re-fit the model. The fitting results of the modified model are shown in Table 7.

**Table 7:** Modified model fit test.

	$\chi^{2}$	df	$\chi^{2}/df$	AGFI	NFI	IFI	CFI	RMSEA	SRMR
**Standard**			<3	>0.9	>0.9	>0.9	>0.9	<0.06	<0.05
**Result**	232.537	96	2.422	0.916	0.924	0.954	0.954	0.055	0.0433

From the modified fitting of the model, $\chi^{2}/df$ is 2.422, AGFI is 0.916, NFI, IFI, and CFI are 0.924, 0.954, and 0954, respectively, RMSEA is 0.055, and SRMR is 0.0433, satisfying the optimal index of model fitting. Table 8 shows the path test after adding control variables. In this model, the live-streamer variables are divided into two categories with corresponding data included. Consequently, the model is fitted.

**Table 8:** Modified model hypothesis test.

Hypothesis	Model after correction		Type1(N = 267)		Type2(N = 196)	
	Estimate	P	Estimate	P	Estimate	P
H1c	0.278	^***^	0.247	^**^	0.289	^**^
H1a	0.546	^***^	0.520	^***^	0.604	^***^
H2	0.229	^**^	0.085	0.383	0.353	^**^
H3	0.511	^***^	0.610	^***^	0.324	^*^

* *p* < 0.05;

** *p * < 0.01;

*** *p* < 0.001.

Type1 refers to the flow live-streaming with a huge fan base; Type2 refers to the official live-streaming with government officers.

After adding the live-streamer type as a control variable, the model fitting test results are shown in Table 8, wherein the hypothesis tests of the modified model are significant. The number of valid samples for flow live-streaming viewers is 267, with significant corresponding hypothesis tests except for H2. Whereas the number of official live-streaming viewers is 196, and the corresponding hypothesis tests are significant.

### Results of hypothesis testing

From Table 6 and Table 8, we can see that H1a and H1c are tested with significance, while H1b and H1d are not significant in the hypothesis that online interaction *via* live-streaming shopping is positively correlated with consumer empathy. The results show that the herd effect and responsiveness of online interaction can largely enhance consumers’ empathy, whereas the effects of mutual assistance and streamers’ image are not significant, which is different from the counterpart results of commercial studies (Jiang et al., 2014).

The findings also show that H2 is not that significant for live-streaming models, indicating that the influence of empathy on consumer impulse buying depends on the type of live streamers. On the other hand, the test of Hypothesis H3 suggests that the streamers’ image is positively correlated with consumer impulse buying.

In summary, the empathy effect acts the role of a partial mediator between online interaction and impulse buying. While adding the live-streamer type as a control variable, the influence of herd effect and responsiveness on empathy effect is greater in official live-streaming than in flow live-streaming. Specifically, the empathy effect of consumers using official live-streaming significantly enhances their impulse buying, whereas the empathy effect *via* flow live-streaming has a very slight impact on consumers’ impulse buying. But inversely, the impact of the live streamers’ image on consumer impulse buying is weaker when using official live-streaming than using flow live-streaming.

## Discussion and conclusion

### Summary of empirical findings

Based on the S-O-R model, this paper has investigated the impact of the live streamers’ image, the responsiveness of sellers, the herd effect of consumers, and the mutual trust amongst consumers on impulse purchases from the dual perspective of live streamers and consumers. We introduce the empathy effect as a mediating variable and the live streamer type as a control variable to explore the underlying mechanism of online interaction’s influence on consumers’ impulse purchases in philanthropic live-streaming marketing. The main findings of the study are listed as follows:

(1) The formation of consumer empathy influences consumers’ impulse purchases positively and significantly. Specifically, the responsiveness of sellers and the herd effect of consumers indirectly influence consumers’ impulse purchases through the empathy effect while mutuality does not affect the formation of the consumer empathy and impulse purchase. On the other hand, the live streamers’ image directly affects consumers’ impulse purchases. Under the control of live streamers’ type, the herd effect and the responsiveness produced by the official live-streaming on empathy is more significant than that by flow live-streaming.

(2) The formation of consumer empathy is positively correlated with impulse buying behaviors, that is, the higher empathy the consumer shares, the greater the chance consumer would purchase impulsively. Impulse buying is an immediate, unplanned purchase behavior of consumers induced by an emotional response to external stimuli, so emotional stimuli play a crucial role in impulse buying behavior. In the context of the pandemic, the philanthropic live-streaming was led by the “Thanks for helping Hubei” campaign with the primary purpose of appealing across the country to purchase agricultural and sideline products stagnantly sold in Hubei, as well as to reduce the negative impact of the pandemic on the local economy. The charitable contributions of live-streaming shopping make consumers feel that they can help the affected people, which encourages consumers’ pro-social preferences and the psychological satisfaction of buying. Thus, the empathy feeling that consumers generate through real-time online interactions with live streamers and other consumers is the central emotional stimulus influencing consumers’ impulsive purchasing behavior.

(3) The responsiveness of sellers and the herd effect of consumers have a strong positive effect on empathy. Consistent with the measurement of the herd effect, which revolves around consumer-to-consumer interaction, a leading slogan such as “Put on weight for Hubei” has achieved the consistency of group opinions that is conducive to raising empathy feelings among groups. On the other hand, the measurement of responsiveness, referring to the interaction between live streamers and consumers using bullet comments, also revolves around issues such as the impact of the pandemic, the need for assistance, product quality, and price. This study shows that intuitive, real-time, targeted, and repetitive feedback will create a sense of responsibility among consumers, which prompts the perception of the recipient’s urgent needs with emotional resonance for supporting agricultural consumption.

(4) The mutual trust between consumers has no significant impact on the empathy effect. Mutual trust between consumers improves the authenticity evaluation of products and purchasing behaviors through the bullet comments in live-streaming. However, this paper shows a weak correlation between mutual trust and empathy, that is, consumer interactions from real-time comments do not affect consumers’ empathic perceptions. This conclusion contradicts the findings from commercial live-streaming shopping. This is particularly because in philanthropic live-streaming, assisting the affected population due to the outbreak of the pandemic is the primary goal of consumers’ participating in the campaigns. Although effective interpersonal interactions can indeed enhance consumer impulse purchases as real-time online comments from consumers can improve the perception and understanding of the actual utility of the products and services and allow consumers to feel like being in a community with warmth, the characteristics of the products (e.g., quality and price fairness) and merchant services (e.g., logistics and after-sales services) are no longer the primary concerns of consumers in the context of philanthropic marketing.

(5) The live streamers’ image positively influences consumers’ impulse buying behaviors but has no significance on the empathy effect of consumers. That means the personal charisma of streamers can directly affect consumers’ impulse buying behaviors without the need to empathize with them. Similar to the celebrity effect, consumers become loyal fans of the streamer during their long-term participation in the live streaming, laying the foundation of the consumer base of the streamer. For instance, Jiaqi Li, a grassroots streamer popular on social media, who is good at product recommendations, has a powerful influence and outstanding consumption conversion rate in live-streaming shopping. Thus, during the COVID-19 pandemic, the streamer’s active participation in philanthropic sales activities can make consumers feel positive toward the streamer’s personality and result in impulsive purchases.

(6) The herd effect of consumers and the responsiveness of sellers have a stronger influence on the empathy effect through official live-streaming than the flow live-streaming. In the context of emergencies, the spirit of leadership is more authoritative and unified in opinion. For example, in the official live-streaming, real-time feedback from local leaders and cadres allows consumers to quickly understand the severity and urgency of the situation, thereby enabling consumers to form empathetic feelings. Whereas in the flow live-streaming mode, the image of live streamers can directly affect consumers’ impulse buying behaviors without the mediating role of empathy. The “streamer+ star” type results in the same outcomes as celebrities, suggesting a better philanthropic live-streaming effect by effectively encouraging the participation of fans.

Different from the majority of existing empirical research on consumer impulse buying behaviors in commercial live-streaming shopping, this paper explores the influence mechanism of online interaction on consumers’ impulse purchases in the philanthropic live-streaming model, which is based on the real case at the outbreak of the COVID-19 pandemic. We also analyze the similarities and differences of how online interaction may influence consumer impulse purchases in both commercial and philanthropic live streaming models. Therefore, on the one hand, this study enriches the empirical analysis of consumer impulse buying behavior in different live-streaming sales contexts. On the other hand, it explores how philanthropic live marketing can promote consumers’ participation in the campaign and enhance sales efficiency under emergencies to alleviate economic hardship.

### Management implications

According to the theoretical conclusions, we further put forward management suggestions on how the philanthropic live marketing model can enhance sales efficiency and alleviate economic hardship under the influence of emergency events from both social and business perspectives.

(1) From the social perspective, the live streamers’ charitable credibility is key to promoting philanthropic live-streaming marketing as the improvement of consumers’ empathy ability and live streamers’ images increase consumer impulse buying. In the face of socio-economic problems such as the stagnant sales of agricultural and sideline products, consumers’ active participation in philanthropic live-streaming sales may largely alleviate the sharp decline in farmers’ income affected by the pandemic. Therefore, it becomes very important to invite streamers with a high charitable reputation to increase the participation intentions of consumers in the campaigns (Monfort et al., 2021; Gao et al., 2022). Streamers and platforms with prior philanthropic behaviors will more likely convince consumers that the current philanthropic sales are credible routines that resonate better with consumers. Therefore, philanthropic live-streaming merchants and resident streamers should normalize their philanthropic behaviors, not only in emergencies such as natural disasters or major public health events but also in regular aid for people with disabilities, children in poverty, and other charitable activities to accumulate credibility and further enhance the influence of streamers.

(2) From the business perspective, philanthropic live streamers should strengthen their effective interactions with consumers. This research shows that an increase in the responsiveness of the sellers is followed by an increase in the empathy effect of consumers, which leads to an increase in active engagement and, in turn, an increase in consumer purchase behaviors. Thus, improving the response efficiency to solve consumers’ questions and strengthening the interactions between buyers and sellers can effectively promote consumers’ purchasing behaviors. Especially, sellers should pay attention to the change in streamer type. As online interaction under official live-streaming can better stimulate the empathy effect of consumers, it is necessary to use a cross-border live-streaming mode, which combines local government officials with traditional streamers, to enhance sales efficiency and produce greater business value through philanthropic live-streaming marketing.

From the perspective of consumers, it is vital to choose a reputational platform for information exchanges since the herd effect of consumers can positively influence consumers’ empathy and thus positively influence their impulse buying behaviors. It is recommended that platform managers should publicize the mission, goal, and vision of philanthropic live-streaming and create a real and comfortable platform environment for consumer-to-consumer interconnection and purchase information sharing.

### Research limitations

Because of the limitations of objective conditions and research capabilities, there are still some shortcomings in this study, which are mainly manifested in the following three points:

(1) The selection of survey samples has limitations. This limitation is mainly reflected in the lack of universality in the selection of sample platforms. This paper mainly uses Taobao live-streaming as an example to conduct research. However, in addition to Taobao, there are live broadcast platforms such as TikTok and JD.COM.

(2) The limitation of research model design. The selection of variables is mainly through literature research, live user interviews, and other ways to summarize the main influencing factors. There is still room for discussion. Whether online interaction will affect consumers’ impulse buying by influencing consumers’ trust and whether consumer trust will affect the formation of the empathy effect remain to be studied.

(3) Limitations of research scenarios. This article mainly carries on the questionnaire survey through the scene pattern. The respondents can only fill in the questionnaire by recalling and associating their Taobao philanthropic live-streaming shopping scene, which may be different from their real purchase situation. For example, the respondents’ emotions at the time of filling in the questionnaire, their quality of memory, and other factors will influence the objectivity of the collected data.

In future research, it is necessary to expand the survey sample further, research consumer groups in different platforms and regions, and conduct a deeper analysis of the online interaction dimension to explore whether there are more influencing factors under the online interaction dimension that will have an impact on consumer impulse buying.

## Data availability statement

The original contributions presented in the study are included in the article/supplementary material, further inquiries can be directed to the corresponding author.

## Author contributions

YY: conceptualization, methodology, data analysis, writing—review and editing, and funding acquisition. ZZ: data collection, data analysis, software, and writing—original draft preparation. HD: software and validation. All authors contributed to the article and approved the submitted version.

## Funding

This work was supported by Sichuan Province Science and Technology Support Program [grant number 2021JDRC0116; 2022NSFSC1865] and Construction Funds for the World-Class University - Integrated Disaster Sciences [grant number 21010144C3001].

## Conflict of interest

The authors declare that the research was conducted in the absence of any commercial or financial relationships that could be construed as a potential conflict of interest.

## Publisher’s note

All claims expressed in this article are solely those of the authors and do not necessarily represent those of their affiliated organizations, or those of the publisher, the editors and the reviewers. Any product that may be evaluated in this article, or claim that may be made by its manufacturer, is not guaranteed or endorsed by the publisher.
